# Using Design-Based Research for a Technological Intervention to Promote Parental Involvement in Kindergarten

**DOI:** 10.1007/s42979-023-01666-8

**Published:** 2023-03-23

**Authors:** Dionísia Laranjeiro, Maria João Antunes, Paula Santos

**Affiliations:** 1 CIDTFF - Research Centre on Didactics and Technology in the Education of Trainers, Department of Education and Psychology, University of Aveiro, Aveiro, Portugal; 2 DigiMedia, Department of Communication and Art, University of Aveiro, Aveiro, Portugal

**Keywords:** Parental involvement, Kindergarten, Technological platform, Design-based research

## Abstract

Parental involvement in preschool education is widely recognized as an important factor in skills development, school outcomes and lifelong success, and can be promoted through digital technologies, which offer new forms of communication and collaboration between parents and educators, and new learning opportunities for children. This research aimed to develop and test a digital platform with communication and content sharing functionalities between parents and educators, and to assess the impact of using the platform in three participating institutions. The research question guiding the efforts was: what features and contents should a multimedia platform have to promote parental involvement in the learning of children who attend kindergarten? The research was developed according to a design-based research methodology, and the parents and educators were involved in all phases of the process: preliminary study, development and evaluation. The results showed that the sharing of activities carried out with children in kindergarten and a private messaging service were the most valued functionalities. Regarding local impact, different results were obtained in each kindergarten, related to the roles assumed by the users within the platform, and previous practices of using technologies for parental involvement.

## Introduction

Parental involvement is a broad topic that involves parents' participation in their children's formal learning process, taking part in school-related activities. Over time, the concept of parental involvement has evolved. Initially, it was assumed as a unidimensional construct, representing the effort and commitment of parents in the child's development, with all activities and initiatives being treated in the same way. It is currently recognized as a multidimensional construct, with different typologies of parental involvement, which have their own characteristics [2] and may be more associated with support for studying, for example, at home or parents' participation in school. Parental involvement is intrinsically linked to the school–family–community partnership concept, whereby the school community, responsible for the student's learning, includes the school, family, and the student himself, as well as other community members who can provide a complementary support according to the school's objectives. When these members form a true partnership, everyone benefits: families are strengthened, schools are improved, the community is more united, and students have more learning opportunities and greater academic success [3]. The importance of parental involvement in children's learning is widely recognized and documented as an important factor in skills development, school outcomes, and lifelong success. Extensive literature reviews and meta-analyses conclude: when there is greater parental involvement, children have better grades, better academic performance, do more homework, are more assiduous, and have stronger study habits and aspirations to proceed to higher education [4–8]. In the school curriculum, there is a relationship between parental involvement and children's grades, ratings, and performance in reading and mathematics [9]. Regarding attitudes and behaviour, parental involvement is associated with fewer discipline problems, better behaviour at home and at school, greater responsibility in decision-making, greater self-control, and more positive attitudes towards school [5, 10–12]. Parental involvement has a significant effect on a child's adjustment to school and learning success, regardless of other factors such as the child's social class, gender, or ethnic group [4]. Furthermore, promoting parental involvement is positively associated with better outcomes for ethnic minority students [13]. Reference [14] also states that involvement can even be harmful, depending on the quality and way in which parents are involved. It can be beneficial if it promotes autonomy, gives confidence and support in carrying out tasks. On the other hand, it can be harmful when it focuses on criticism, or on the child's difficulties in carrying out tasks, controlling and putting pressure on the student to make decisions.

At preschool age, it is associated with general development, social and cognitive development, preparation for school, and the development of literacy skills [15] and math skills [16]. It is in preschool education that children benefit most from parental involvement in learning, whether at home or in kindergarten [17]. Kindergarten is an inviting environment for parents to participate. They are effective in the help they can provide and are motivated to give their children a good start in schooling [18]. For this age group, several authors propose the division into dimensions of parental involvement [17, 19]. In this study, we opted for the typology of Ref. [20]. The authors developed the Family Involvement Questionnaire (FIQ), an instrument to assess different dimensions of parental involvement in kindergarten, which was developed and tested with parents and educators. This instrument has been used in research for over a decade and has established three dimensions of parental involvement: involvement at home (active learning with the family); involvement in the school/institution (collaboration in kindergarten activities); and family communication with kindergarten (contacts about the child's progress). Over a decade, the FIQ has been used in preschool education in several research projects, showing consistency and invariance in dimensions, across different regions, languages, genders, social classes, and ethnicities [20]. The importance of parental involvement is recognized in government guidelines for preschool education in several countries [21].

Children at these ages learn essentially in the restricted and immediate environments in which they live—the family and kindergarten [22]. Portugal has curricular guidelines for preschool education that give autonomy to kindergarten teachers, in their pedagogical activity and choice of methodology [23]. Factors such as the individual characteristics of the children, the size of the group, or the diversity of ages will influence the group's functioning, the pedagogical options, the projects developed and, finally, individual learning. All these variables make it difficult for parents to know what their children learn in kindergarten, which may reduce their active participation in this process. Good communication between kindergarten and family contexts can improve the knowledge they both have about the child, influencing learning [24]. For kindergarten, communication with families is important to gather information about children and build an adequate curriculum, a stimulating environment, and meaningful learning [23]. For parents, the knowledge they have of what their children learn in kindergarten allows them to more easily think and carry out activities and games together, creating quality moments while encouraging the child to build knowledge.

Currently, the Internet and digital tools are part of families' lives. In kindergarten, digital media bring new learning opportunities for children and new forms of collaboration and transmission of information between parents and educators [25]. Digital technologies can help to overcome some barriers to parental involvement, such as lack of time, schedule conflicts, and availability [26].

In addition, several studies indicate contributions of digital technologies in children's learning, in terms of language development, mathematics, knowledge of the world, multiliteracies, creativity, arts, motivation, and collaborative learning [27, 28]. The widespread access to mobile devices and educational apps by preschoolers has brought them new opportunities and ways of understanding, acquiring knowledge, and expressing themselves [29], although parents and educators struggle to identify apps with real educational value [30, 31]. Thus, a digital platform can also serve to share interactive educational content for learning activities with children.

The current COVID-19 pandemic has led countries around the world to close schools and implement distance learning solutions to reduce contamination. This situation has shown the need to improve communication between parents and teachers by digital means and provide educational content online for all ages [32].

Access to appropriate technological devices and digital content can help parents to promote their children's learning at home. Using social web tools and private communication platforms, parents and educators can share information about their educational practices. Educators can form virtual groups that encourage parents to participate in kindergarten and in their children's learning. Children can be involved in these dynamics, to acquire knowledge and develop skills, such as communication and collaboration with adults and other children.

This chapter is an extended and revised version of the paper “An Intervention with Technology for Parental Involvement in Kindergarten: Use of Design-based Research Methodology” [1], presented at CSEDU 2022. It is divided into the following sections: “Introduction”, “Methodology”, “Results”, “Discussion”, and “Conclusions, Limitations and Future Studies”. The main differences from the previous article are the following. In the introduction, the theoretical framework is extended to better explain the concept and importance of parental involvement, different perspectives, and dimensions, which are later used in the results. As an article that intends to demonstrate the application of design-based research, “Methodology” was also increased, to better address the distinctive characteristics of the methodology, types of objectives, and data collection and techniques of data analysis. The operationalization model is described in more detail. In “Results”, the presentation of results achieved in each phase was increased, which was not possible in the previous article, due to text size limits. Thus, in the preliminary study, statistical data from the questionnaires to the parents are presented to better understand the technological affinity of the participants, and some of these data were combined with the content of the interviews with the educators. In the development, the planning process, the platform's features, and the different profiles and access permissions are further described. In the evaluation, data from web statistics are also presented, which were essential to understand accesses and visits to the platform during the pilot study, cross-checking the data (content published on the platform and surveys to the participants). It has a wider and deeper discussion of the design principles that emerged from the research as well as the results of the impact on each classroom and the limitations of the study. Eighteen new references were added, to include authors, theories, and research relevant to the themes addressed in the chapter.

## Methodology

This research aimed to plan, develop, and evaluate a multimedia platform, to answer the question: what features and contents should a multimedia platform have to promote parental involvement in the learning process of children attending kindergarten?

From this research question, two types of responses were expected: (1) a general contribution to the theory—design principles that can be applied in educational interventions in similar contexts; (2) a local contribution, related to the impact of using the platform on the parental involvement of a group of participants. The research team collaborated with the technological team of a multimedia company. Four kindergarten classrooms, 4 educators, and 94 parents participated, collaborating in all phases of the project: definition of the platform; prototype testing and use; final evaluation of the platform (as a technological product); and evaluation of the intervention (impact of use).

Ethical and privacy issues were assured during the research. Three kindergartens were identified and invited to participate in the project, having as a requirement the predisposition to use technologies with children and parents, as this would be an essential condition for using the platform and collecting useful information for planning, development, and evaluation. Participants received information about the project, goals, expected results, and their expected participation. They gave informed consent and volunteered to participate. Data collection were in accordance with GDPR and ensured anonymity and pseudonymization. The treated data were available to participant’s analysis, guaranteeing accuracy and transparency.

The design-based research (DBR) methodology was adopted, considering the characterization of the problem, the objectives, the research question, the context, and participants in the study. DBR is used in interventions to solve a complex educational problem and at the same time improve knowledge about the development process and characteristics of the intervention [33]. The intervention can include technological prototypes, content, and environments that use technology, with a potential impact on teaching and learning. DBR is not directly related to information and communication technologies, although it is a methodology widely used in the field of technologies in education and instructional design. It allows exploring the potential of digital technologies in education, to solve a real problem [34]. The development process is iterative, consisting of cycles of analysis, design, and evaluation, until reaching a satisfactory approximation of an ideal intervention. Reference [35] adds that the DBR is developed in a real educational context; therefore, the results are used to improve local practices and evaluated to inform theory. The context must be carefully characterized, as the design principles that emerge must reflect the conditions of the intervention [36]. The intervention should include collaboration between researchers, professors, users, and experts, who work together to better align the research process and results with the needs and expectations of society [37], which is a condition for research and responsible innovation (RRI). DBR combines qualitative and quantitative techniques for data triangulation and validation of results, at different stages of development [36]. Some examples are literature reviews, interviews and questionnaires, Delphi techniques, think aloud protocols, observation, and document analysis [36, 38]. The diversity of qualitative and quantitative data can help to illustrate, give credibility, and present a more complete picture of the research [39], although there is a greater tendency to use qualitative techniques to understand the complexity of real situations [38]. This type of research brings context-specific knowledge, but it may be transferable and relevant to other learning environments [40]. According to Refs. [41] and [42], the knowledge resulting from the DBR is usable, adaptable, and adoptable. Adoption requires interpretation of the context in which it was carried out, so that the adaptation is successful. Therefore, it is necessary to be well documented, to share the product, and provide rich descriptions of the context, explain the theory, the particularities of the intervention, and the impact of the intervention on learning [43].

For all these reasons, the DBR methodology was chosen for the development of this project. The platform was built to modify a specific situation, which was to increase parental involvement in learning using technology. There was a continuous collaboration of researchers with the technological team, kindergarten teachers, and parents, who were involved in all phases of the project. The development of the platform was interactive and iterative, that is, the platform was used and evaluated in context, corrected, modified, and enhanced to improve the intervention, in three development cycles. A combination of qualitative and quantitative techniques was used for data collection and analysis at different stages.

For this study, [33] DBR operationalization model was adapted, dividing the research into three phases: preliminary study, development, and evaluation. In each phase, a combination of data collection techniques was used, according to different objectives.

In the preliminary study, the following procedures took place: characterization of the context, using data from interviews with educators and questionnaires to parents; literature review for theoretical contextualization; web search to identify and classify existing platforms on the market; survey of educators' needs, through interviews; survey of parents' needs through questionnaires.

The development of the platform was divided into three iterative cycles of analysis, design, and formative evaluation, until reaching the final product. In the first cycle, the functional specifications were defined, and a paper prototype was developed. UI–UX [user interface–user experience] tests allowed collecting data for a first evaluation. In the second cycle, a functional prototype was developed, which was used by educators and parents in a pilot implementation in kindergartens. Questionnaires to parents and interviews to educators served to understand parental involvement practices of the participants. To monitor access to the platform, an analytics software was used to collect data. To monitor user participation on the platform, the content of the posts was analysed. To collect feedback from participants, e-mails, meetings, and research notes were used. Data analysis from this phase led to a mid-term assessment before the last development cycle. In the third cycle, the development of the platform was completed, and it was used in kindergartens until the end of the school year. The same techniques were used to collect data on access to the platform, user participation and receiving feedback from participants.

The final evaluation served to evaluate the practical results of the intervention, the platform's impact on parental involvement in children's learning, and contributions to theory with design principles and suggestions for future studies. In this phase, we analysed: the content published on the platform (collected from the posts database); visits and accesses to the platform over time (collected by analytical software); content of interviews with educators and focus groups with parents, to collect their perception on the use of the platform.

## Results

### Preliminary Study

The preliminary study aimed to characterize the context and understand the needs of parents and educators regarding the features, contents, and dynamics that a platform for parental involvement should have, to include both perspectives in the development. At the same time, it was intended to understand the state of the art of platforms already on the market and to know the strengths and weaknesses, good practices, and gaps to be filled. The preliminary study consisted of questionnaires to parent (*n* = 59), interviews with the four educators (Ed1, Ed2, Ed3, Ed4), survey of platforms available on the market (*n* = 12), and the literature review. The data collected in these four components of the study were analysed and cross-referenced to help understand the most important features to be developed in the future platform, the advantages perceived by users and the possible constraints to its use. The results also helped to characterize the context. Parents mentioned using the Internet (100%), daily (88.1%), on the computer (96.0%) and mobile phone (96.0%). The computer was most used for web search (89.8%), e-mail (81.4%), file sharing (78.0%), and purchasing goods and services (72.9%); and the mobile phone was most used for e-mail (76.3%), instant messaging (71.2%), and social networks (62.7%). These results are in line with data from the National Statistics Institute [44] on the growing trend of access and widespread use of the Internet by Portuguese families. Their children also accessed technology at home, especially the tablet (76.3%) and the Internet (62.7%). Parents used technology to do activities with their children (84,7%), such as filming and photographing with the child on the cell phone (72.9%), showing videos and photos to the child on the mobile phone (66.1%) and on the computer (61.0%), using games and educational apps on the tablet (57.6%) and doing research on the Internet on the computer (54.2%). When asked about the use of technology in kindergarten, parents only mentioned the computer (45.8%) and the Internet (8.5%). On the contrary, all the educators mentioned that they used computers and Internet daily, for personal matters and teaching activities with the children (“Search (web)… around a topic we are working on” (Ed1) and allowed the children to use the computer independently (“Inside the classroom, we have several areas and one of the areas is the computer. They can go there to play and work.” Ed3). These diverse answers seem to indicate that parents were not aware of the digital learning activities carried out in kindergarten. From this part of the study, it was concluded that the group had good technological affinity, a favourable condition for the planned intervention.

Regarding features, on a scale of importance from 1 to 5, the features most valued by parents were: news and events schedule (both with an average of 4.52), photo and video gallery (average of 4.48), and a private messaging service with the educator (average 4.25). These were also the features most found on existing platforms on the market. The educators agreed with the parents about the most important features, but considered that the platform should also gather the parents' contacts, the children's history (“the entire history of the child, whether in terms of health or in terms of evolution, records, assessments we do” Ed2), and function as a social tool to encourage parents to share suggestions for activities and links to digital educational resources (“it would be fun to be something more interactive. We [educators] could post the activities we do with the children and they [parents] could comment.” Ed4). The existing platforms, which were more suited to the kindergarten context, focused on disseminating information about the institutions' activities, but did not provide strategies or suggestions to parents, who could contribute more actively to their children's learning. Both parents and educators pointed out that an advantage would be the platform providing information to parents, helping to start conversations with children about what they learn. These aspects are highlighted in the literature: a digital platform can inform parents about what their children are learning, guide parents in creating new learning opportunities at home, and involve parents in distance activities with kindergarten [45]. Also, as advantages, parents considered that the most important thing is access to updated information about activities carried out in kindergarten. The educators mentioned the automation of communication and the promotion of parental feedback. These advantages are also the most reported in the literature [46]. Another advantage identified by the educators was the separation of personal and professional social media (“When parents invite me on Facebook, I accepted it, but that was my personal space, and now it is mixed with professional matters” Ed2). Regarding constraints, parents expressed a general concern about the protection of personal information, in particular, the sharing of photographs where children were identified, and 66.1% considered that only the educator and parents of a classroom should have access to information about that group, excluding assistants, directors, and parents from other classrooms of the institution or guests. Educators indicated the lack of time to update information on the platform (“As I am also a pedagogical coordinator, it is difficult for me to find time for everything.” Ed3) as well as the non-adherence of parents or misuse (“If people start using it to talk about things that, perhaps, are not of interest to the community.” Ed4). An in-depth presentation of the preliminary study is available in Ref. [47].

### Development

The preliminary study helped to understand the most important features, dynamics, and content for parents and educators in a parental involvement platform for kindergarten. Based on the contributions of the preliminary study, the second phase of the research began—the development of the platform. This phase was divided into three cycles. In the first cycle of development, the functional specifications were defined, and a paper prototype was drawn up for a first formative evaluation with users. It started with the creation of the architecture diagram and definition of the conceptual framework. This was followed by a more detailed description of each functionality, user profiles, and access levels. It was defined that the platform would have a group area, for communication and information sharing between the educator and parents of children in the same classroom; a personal area, for private communication between educator and parent (1:1); an institutional area, with unidirectional communication from kindergarten to parents. Public areas were excluded, respecting the apprehension shown by parents and educators in the preliminary study. Three profiles were defined: administrator, educators and parents. The administrator is responsible for managing the platform, creating groups and members, and accessing statistical information and content for research. Educators have read/write permissions in the group area and in the kindergarten area. In the private area, they can read/write the history of all the children in their classroom. Parents can read/write in the group area and read in the kindergarten area. In the personal area, they only access private information about their child.

A paper prototype, simulating the main pages of the platform, was designed to test usability at an early stage of development, when it is easier to introduce changes and improve the user experience [48]. It was submitted to tests with parents and educators, to collect information from the two profiles. At this stage, the topics to be evaluated were the relevance of the content, the consistency of the design, and the expected practicality, that is, whether the product was expected to be used in the context for which it was created, or not [36]. The tests were carried out by the researcher with four educators and four parents, and they followed the same procedures. Individually, users looked at the first screen and described what they saw. Then, they “walked-through” the screens, performing tasks requested by the researcher (e.g. “see if you have new messages”), while users “thought aloud”, commenting on the tasks they were doing. At the end, an interview was carried out to understand the attitudes and expectations regarding the future use of the platform. The evaluation with users allowed to verify the general understanding of the project by both profiles and to identify some improvements and changes to the initial prototype: create new areas (edit profile, personal page, meals), merge different areas into one (events and agenda; documentation and information), simplify the field of writing comments, and present contents in chronological order (links, agenda, and activities).

**Fig. 1:**
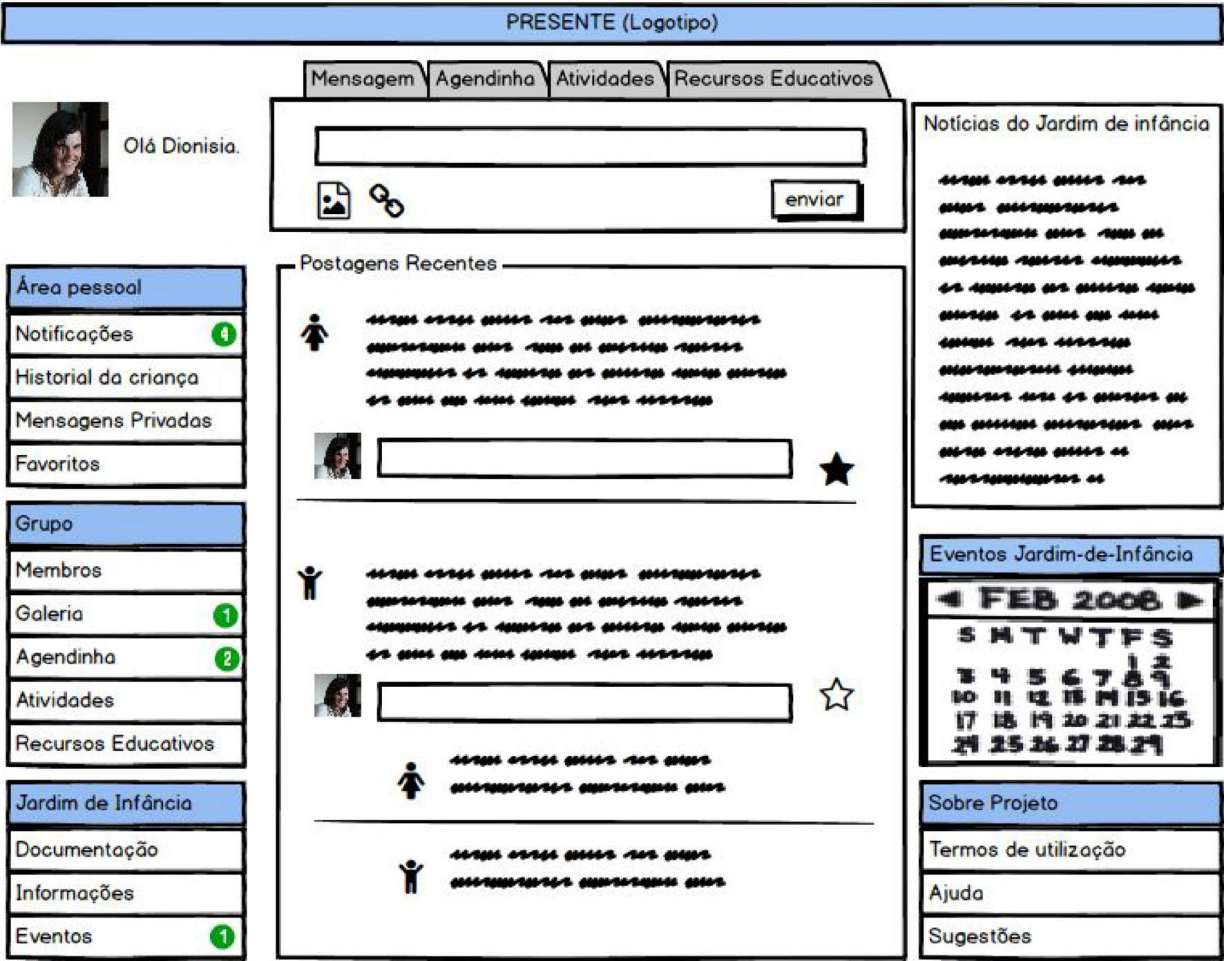
Paper prototype [1]

Figure 1 shows the main page of the paper prototype tested with users. In the left side menu, the user accesses the three areas: personal (“Área pessoal”), group (“Grupo”) and institutional (“Jardim de Infância”).

From the interviews, it emerged that users valued the platform. The educators intended to use it daily to share activities with parents, while parents assumed a weekly use, more oriented towards communication with the educator than to sharing information with other parents, or to carrying out educational activities with their children. In Ref. [49], all procedures and test results with users are presented, as well as the structure and functionalities defined for the platform.

In the second development cycle, a functional prototype was developed with selected features presented in Table 1:

**Table 1:** Data collection in each phase

Personal area	
Child history	Sharing information about the child between parents and educator (1:1)
Favourites	Inform when there are new posts
Notifications	Web search
Edit profile	Edit personal information
Group area	
Activities	Sharing suggestions for activities, sharing activities done in the classroom
Events	Sharing of educational events, sharing events from kindergarten
Educational links	Sharing of educational sites, apps and other digital resources
Kindergarten area	
News	Institutional news shared by the educator

**Fig. 2:**
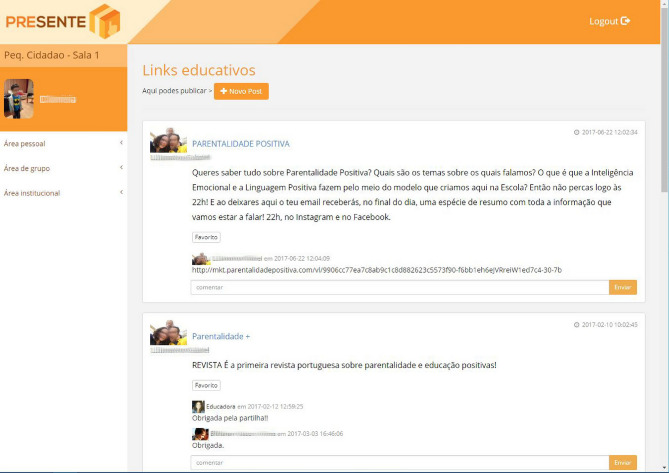
Functional prototype

The prototype (Fig. 2) was used in a pilot implementation, with 3 kindergarten classrooms (KG1, KG2, KG3), 3 educators, and 67 parents. The fourth kindergarten classroom dropped out because the educator was on maternity leave.

The pilot study began, with meetings in kindergartens, to present the platform to the participants. Questionnaires were distributed to parents and interviews were carried out with educators, to inquire about the practices of parental involvement prior to the intervention. From the interviews with the educators, it was concluded that they were all active in parental involvement, but had different technological strategies. Ed1 used e-mail and created a weekly digital newsletter, which was posted online and shared with her classroom parents. Ed2 used multiple digital media for parental involvement: a private Facebook^®^ group, e-mail, Messenger^®^, a cloud service for sharing photos, and Skype^®^ for video calling. Ed3 only used e-mail occasionally.

**Fig. 3:**
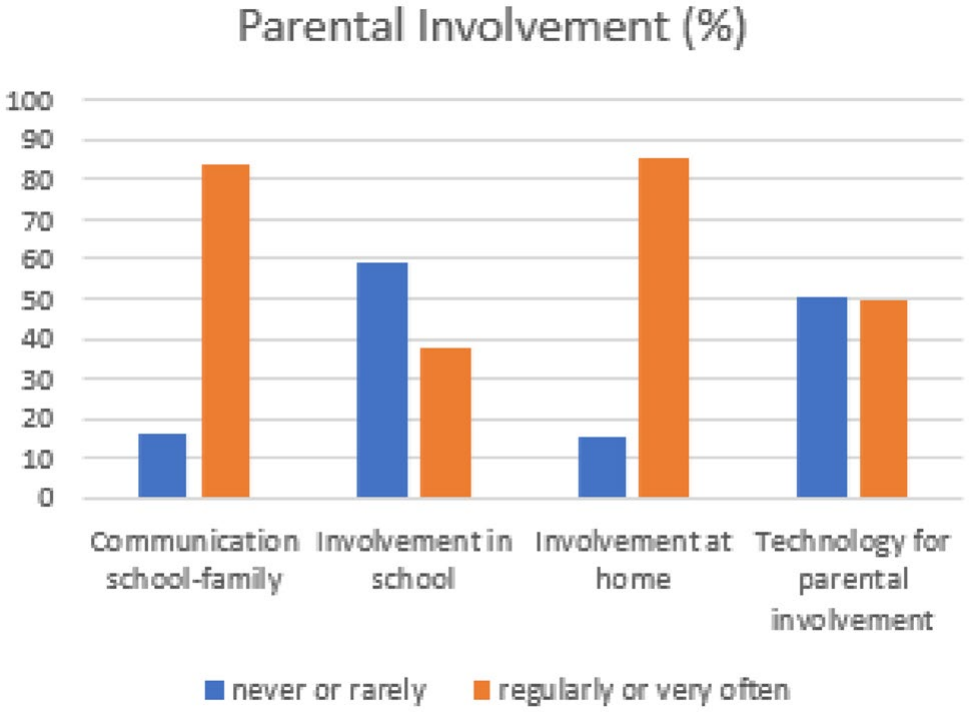
Participants’ parental involvement chart [1]

Parents answered a questionnaire (*n*=45), with the three dimensions of parental involvement—involvement at kindergarten, involvement at home, and communication with the educator. It also questioned about the use of technology for parental involvement. It was concluded that parents essentially valued the dimensions of communication with the educator and involvement at home. Digital technologies were most used in parental involvement at home (Fig. 3). Thus, the platform, which was designed to facilitate these aspects, was well positioned to be adopted by parents.

During the pilot study, the researcher followed the evolution of the platform's use. Visits and accesses were monitored through a web statistics program. User posts collected on the platform were analysed using content analysis to systematize qualitative data according to the frequency of occurrence of certain terms and text meanings [50]. Data analysis was performed using the NVivo^®^ software, which helped to organize, transcribe, code, order, and categorize the data. The unit of analysis was the sentence excerpt from the posts, which was associated with a topic represented in a coding category. After coding, NVivo® indicates the units of analysis associated with each category, subcategory, and node, giving an overview of the expressiveness of each topic. Feedback received through periodic contacts with educators (e-mails, phone calls and meetings) and parents (e-mails) allowed to fix bugs in the platform and identify improvements that were implemented in the last development cycle, such as online security measures and the inclusion of image galleries.

The pilot implementation ended with interviews with educators (Ed1, Ed2, Ed3) and two focus groups with parents (*n*=15; *n*=5) to obtain more in-depth information about their use of the platform.

### Evaluation

The final evaluation aimed to verify the practical use and effectiveness of the intervention, that is, whether the platform was used in the context for which it was developed and served to achieve the expected results [33] to promote parental involvement in the learning of children in kindergarten. In the final evaluation, web statistics, the content published on the platform, and the content of interviews and focus groups were analysed.

Using the web statistic program, it was possible to monitor the evolution of the number of visits and page views on the platform over time.

**Fig. 4:**
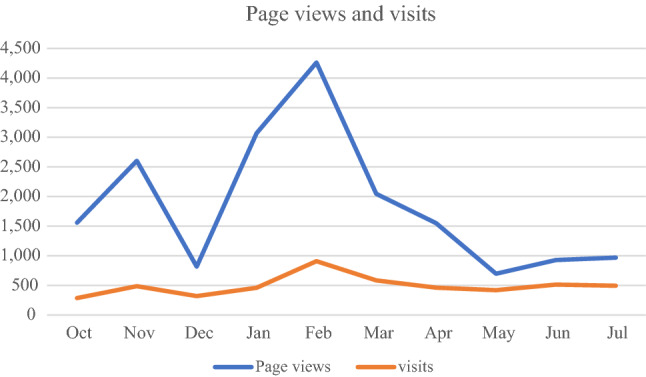
Web statistic chart

From January to March, the platform had the highest values of visits and page views, which correspond to the period of greatest publication of content on the platform (Fig. 4). Crossing the information collected by the program with the published content and the results of user surveys, it was concluded that the peaks of access to the platform were caused by the dynamization within the platform, the publication of new features, and the insistence of the educators on parents' meetings.

Communication and interaction were different in the three kindergartens. There were also considerable differences in the two profiles (parents and educators). In KG1, there was a high amount of communication in all directions (between parents, parent–educator), initiated by the educator or parents (proactive), or in response to comments (reactive). Parents shared events and proactively created photo albums. In KG2, there was no communication between parents, only between parents and educator, always initiated by the educator, with parents replying to comments. In KG3, there was communication in all directions, but reduced. Parents proactively shared links to articles on education and parenting and replied to comments from each other and from the educator.

The educators were the main drivers of the platform. They posted 46 activities, 23 links, and 15 events and responded to 9 comments from parents (e.g. "He has been very attentive to the world. So attentive he even needs a magnifying glass."—Ed1 "). Parents took on different roles—40 remained observers (no participation), 31 responded to messages/posts (reactive participation), and 10 started new conversation topics (proactive participation). The web access statistics were high (4,935 visits in 10 months), which seems to indicate that the parents took a passive role on the platform, perhaps because their goal was just to visualize information, or because they needed time to become familiar with a new social tool [51].

The areas with the highest number of publications were: activities (48), where educators shared activities carried out with the children, encouraged parents to participate in kindergarten, and to publish on the platform; links (34), where users essentially shared videos, links to photographs, and educational articles; events (18), where they shared kindergarten events, leisure events, and educational events.

Parents' comments had varied content: they added information about the child (36 comments), (e.g.: “he is very stubborn, he never wants help.”), they added information about activities at home (10 comments) (e.g.: "He's been reading this story a lot... Why do you have such big ears? It's to hear you better!"), feedback (25 comments), greeting (22 comments), general information (7 comments), technical questions (8 comments). Some comments denoted great enthusiasm and satisfaction (6 comments) (e.g. “Sooooo gooooood!!! Mom loves your kisses too :) Good job!!!”); and complicity with the educator (13 comments) (e.g. “Love is in the air (Ed3) - “It's normal it's spring… And on top of that the educator is always fostering marriages”). Comments about the child and comments about activities at home or kindergarten have the greatest influence on learning, as they provide information about the contexts, which educators and parents can use in learning [25]. The other types of comments are also important to maintain active and positive communication and establish a climate of trust for long-term relationships [52]. It can be concluded that the platform promoted parental involvement, in the dimension *school–family communication*, because it generated communication and content sharing about children's learning between parents and educators.

The interviews and focus groups made it possible to know the perception of educators and parents about the use of the platform. Some results are summarized. The parents' reasons for accessing the platform were the sharing of activities carried out in kindergarten, interesting games proposed by the educators, and the insistence of the educators. The features considered most useful were those that promoted group sharing—educational links, events, activities, and photo gallery. Regarding the inclusion of children in the project, six mothers said they used the platform with their children, to show photos and talk about the activities, which means that the platform generated parental involvement, in the dimension *involvement at home* (e.g.: "yes, we talked informally, how was it, if she liked it, if she didn't… the conversation flowed and that was good”).

Both profiles suggested improvements for the future, in particular, the possibility to manage notifications and better usability on mobile devices. The perceived advantages of the platform were the immediate sharing of information about the daily lives of children in kindergarten, promoting more continuously online school–family communication (“I think they (parents) end up having a more trustworthy portrait of what our day-to-day is. I think that's where it contributed the most.”—Ed1). The constraints mentioned were the lack of time and excess work that the educators already had, the dynamization being centred on the educators, some technical difficulties and, in the case of KG2, the fact that they already use other communication tools.

## Discussion

This research proposed to achieve two types of contributions: a general contribution—design principles of the intervention and the platform; and a local contribution—the impact of using the platform on the involvement of parents participating in a local intervention.

The information generated in the three phases of the research, the participation of three kindergartens, educators, and parents with different parental involvement strategies and different technological uses, the triangulation with theoretical studies and other existing platforms, allowed the creation of guidelines for the design of a technological intervention in similar contexts. For this purpose, only the results that were constant for the three classrooms and mentioned throughout the three phases are retrieved, in relation to the features, content and dynamics that can be promoted with the platform for parental involvement, as well as other indications that stood out for the success of the intervention.

The most valued features are those that allow the sharing of activities carried out with children in kindergarten, whether it is a chronology of posts or an image gallery. Others were also used, mainly the sharing of events and links. Another feature often mentioned as necessary was notification of new content. This topic was mentioned in all phases of the study by the two profiles. It is important that users receive notifications when there is new content, to remind them to access the platform. However, notifications can become a problem in a very active group, so a notification management mechanism should be provided, in which the user decides the information to be notified. In terms of content, parents mainly wanted to see their children's activities and know how they spent their day. In addition to these contents, they also shared other types of information—family events and educational articles, among others. Therefore, it is important to have areas with sufficient flexibility to integrate the different interests and contents.

The dynamization depends essentially on the educators. They played an important role in e-moderating, releasing new content, encouraging participation, and replying to comments from parents. If educators do not assume this role, participation may be residual. The educator must be able to set aside time for this task, to think about new dynamics, insert content (texts, photographs, links), and respond to parents. It is essential that the platform is easy to use, with quick content insertion (for example, uploading multiple images to the gallery at the same time), and without many mandatory fields. Private messages must allow simultaneous sending to more than one user. Parents can take on different roles—passive observers, or reactive or proactive participants. This is because their interests are also different. Some parents just want to receive information about their children, others want to communicate with the educator, and a smaller group likes to share content with other parents. The group itself and its previous relationship can influence participation, and for this reason, the platform must be prepared for different types of communication (one-way, two-way, and multi-directional). In this regard, post commenting functionality and private instant messaging are possible options. Due to lack of time, the institutional area was not updated by educators, although it was considered important at all stages that participants were surveyed, so it seems that an administrative profile could be useful to update information, such as cafeteria menus, events and kindergarten news. Mobile access seems to be a condition for more frequent use, so the platform must be optimized for these devices, in terms of ease of access (login), reading, writing and publishing content. The privacy and security of information must be guaranteed and explained so that parents feel safe to join and participate in the platform.

Regarding the local impact, the three cases (KG1, KG2 and KG3) had different results, which may be explained by the different strategies of parental involvement with technology that each educator had previously.

Before the pilot implementation, the KG1 educator was already using technologies for parental involvement, in particular, a weekly newsletter created by herself, on a web page where she added texts, images and links, and then sent to parents by e-mail. However, creating the newsletter was a lot of work and the educator wanted a more automatic way to communicate with parents and receive feedback, so there was a good predisposition to use the platform. In this group, during the pilot, there was an intensive use of the platform. The educator used the platform to create games and challenges for parents and she shared the activities carried out with the children, as well as photo albums and educational links. The parents responded to the educator and to each other, generating communication in all directions. Parents' comments expressed appreciation, feedback and greetings. Some added information about their children or about the activities they did at home. Parents also took responsibility for updating some areas, such as educational events and galleries to share photos of the children. Parents mentioned that they used the platform to share photos and because they were curious to see what their children had done, but they were also interested in the educator's dynamization, who involved them in experiences and games. Both the educator and the parents considered that the platform played a role in the parents' understanding of the work carried out in the kindergarten regarding their children's learning. The educator did not use the platform with the children, but some parents shared the published content with their children and talked about it, thus serving for parental involvement at home. The use of the platform at KG1 fulfilled its functions as a tool for parental involvement, in the dimensions of school–family communication and parental involvement at home.

At KG2, the parents and the educator were already using various digital communication tools regularly, such as a Facebook^®^ group, Skype^®^, and Messenger^®^, Cloud service to share photos and e-mail. For this reason, they made many suggestions in the preliminary study to define the platform, considering it advantageous for bringing together all the features they needed in a single space. However, during the pilot implementation, the educator shared publications on the platform, but the parents did not participate, and continued to use the tools they already used before. In addition, the fact that the prototype was still under development and needed adjustments and corrections, discouraged its use by a group that already had its digital dynamics established. In conclusion, the platform did not replace previous technological means, because users did not feel that need. In this group, an experience was carried out, including the children in the dynamization of the platform. Children shared their drawings and videos, which resulted in the parents' punctual and intense use of the platform to see and comment their child’s activities. In this dynamization, the platform promoted parental involvement at home and school–family communication, briefly fulfilling its function, but was not adopted in the long term.

In KG3, there were no previous habits of using technologies for parental involvement. The educator occasionally used e-mail, preferring face-to-face contact, and posting the children's work on bulletin boards near the classroom. Some parents had already suggested the use of a platform for communication, which is why the institution decided to join the project. During the pilot, the educator made a great effort to dynamize the platform and obtained little participation from parents. Some parents shared educational articles and responded to comments from the educator and other parents. The educator also reported some technical difficulties, which together with the low participation of parents and the need to duplicate information by traditional means generated frustration and a residual participation at the end of the pilot. However, at the beginning of the new school year, the institution contacted the researcher, as the parents wanted to use the platform again. Five new virtual rooms were created for the institution, not only for the kindergarten, but also for the day-care centre. In this kindergarten, the platform did not have a major impact on parental involvement during the pilot implementation, but it did have an impact as a way to raise awareness of the need to use technology for these purposes. Thus, the intervention came to change an educational situation with a technological product, which is the purpose of DBR.

## Conclusions, Limitations and Future Studies

This research intended to answer the question—what features and contents should a multimedia platform have to promote parental involvement in the learning process of children attending kindergarten? As already presented in the discussion, we concluded that the main features, both for parents and for educators, are those that allow the sharing of activities carried out in kindergarten, either in a group (all parents from the classroom) or privately (between parent and educator). Regarding content, parents want to see their children's projects and receive information on how they spent the day, which can be in images or texts. In the three kindergartens, the results of using the platform were different, but it was possible to verify its contribution in promoting parental involvement, in terms of kindergarten-family communication and parental involvement at home.

The limitations found in this research are typical of the methodology. DBR involves several people with different profiles and rhythms—researchers, technological team, and users [53]. The research required time to collect and analyse the data at various stages. The technological team had reduced availability, due to the reconciliation of several projects simultaneously. Educators and parents were conditioned by schedules, school calendars, and personal availability.

A limitation of the project was the impossibility of matching the availability of the technological team with the research phases in which empirical results were obtained to apply in development, so some suggested improvements were not implemented on the platform. These restrictions limited technological development, which may have influenced the results on the impact of the platform for parental involvement.

DBR is long, due to its cyclical and iterative character [35]. As technology evolves rapidly, DBR can take a long time to respond, so cycles should be brief. The organization by phases (preliminary study, three development cycles, and evaluation) implied the determination of a schedule with rigorously defined deadlines. This resulted in a short pilot period, for users who needed time to appropriate social tools, to adapt to this new form of communication and interaction, and to create access and participation routines [51, 54]. The case of KG3 is an example of this need, because during the pilot implementation, the parents did not have much participation, but in the following year they asked to use the platform again. A study on the evolution of the use of the platform in consecutive years in this kindergarten would be interesting.

Another limitation is the difficulty in generalizing the results. It is not possible to use representative samples of reality in software development, as it would be necessary to analyse large amounts of data generated between development cycles. Even with small samples, it is difficult, due to the variety and amount of data generated and triangulated in all phases [40]. Thus, the products are tested in small groups and launched on the market. Later, with continued use and new data, they evolve into optimized versions.

For the future, it will be necessary to make some changes to the platform, to solve the constraints on its use, to be adopted in other kindergartens, where it can contribute to parental involvement.

## Data Availability

The datasets generated during and/or analysed during the current study are available from the corresponding author on reasonable request.

## References

[1] Laranjeiro D, Antunes MJ, Santos P (2022). An intervention with technology for parental involvement in kindergarten: use of design-based research methodology. Inter Conf Comp Supp Edu..

[2] Grolnick W, Slowiaczek M (1994). Parents’ Involvement in Children’s Schooling: A Multidimensional Conceptualization and Motivational Model. Child Dev.

[3] Epstein J, Salinas K (2004). Partnering with Families and Communities A well-organized program of family and community partnerships yields many benefits for schools and their students. Edu Lead.

[4] Desforges C, Abouchaar A (2003). The impact of parental involvement, parental support and family education on pupil achievements and adjustment: A literature review. Nottingham..

[5] Henderson A, Mapp K (2002). A new wave of evidence: the impact of school, family, and community connections on student achievement. Annual Syn..

[6] Henderson A, Berla N (1994). A new generation of evidence: The family is critical to student achievement. National Com Cit Edu..

[7] Jeynes W (2005). A meta-analysis of the relation of parental involvement to urban elementary school student academic achievement. Urban Edu.

[8] Jeynes W (2012). A Meta-Analysis of the efficacy of different types of parental involvement programs for urban students. Urban Education.

[9] Sonnenschein S, Stapleton LM, Metzger SR (2014). What parents know about how well their children are doing in school. J Educ Res.

[10] Epstein J (1995). School/family/community partnerships. Phi Delta Kappan.

[11] Harris A, Goodall J (2008). Do parents know they matter? Engaging all parents in learning. Edu Res.

[12] Melhuish EC, Phan MB, Sylva K, Sammons P, Siraj-Blatchford I, Taggart B (2008). Effects of the home learning environment and preschool center experience upon literacy and numeracy development in early primary school. J Soc Issues.

[13] Jeynes W, Din M (2021). Parental involvement for urban students and youth of color. Handbook of urban education.

[14] Pomerantz EM, Moorman EA, Litwack SD (2007). The how, whom and why of parents’ involvement in children’s academic lives: more is not always better. Rev Educ Res.

[15] Skwarchuk SL, Sowinski C, LeFevre JA (2014). Formal and informal home learning activities in relation to children’s early numeracy and literacy skills: The development of a home numeracy model. J Exp Child Psychol.

[16] Susperreguy MI, di Lonardo Burr S, Xu C, Douglas H, LeFevre JA (2020). Children’s home numeracy environment predicts growth of their early mathematical skills in kindergarten. Child Dev.

[17] Reynolds AJ, Shlafer R, Din M (2010). Parent involvement in early education. Handbook of school–family partnerships.

[18] Stevenson DL, Baker DP (1987). The family-school relation and the child’s school performance. Child Dev.

[19] Weiss H, Caspe M, Lopez ME (2006). Family involvement in early childhood education. Har Family Res Project..

[20] Fantuzzo J (2013). Multiple dimensions of family engagement in early childhood education: evidence for a short form of the family involvement questionnaire. Early Child Res Quarter.

[21] European Commission/EACEA/Eurydice/Eurostat. 2014 Key data on early childhood education and care in Europe, Luxembourg.

[22] U. Bronfenbrenner, 1979 The Ecology of Human Development Experiments by nature and design. Cambridge, Massachussets and London: England: Harvard University Press. USA

[23] Silva IL, Marques L, Mata L, Rosa M (2016). Orientações Curriculares para a Educação Pré-Escolar.

[24] J. Epstein, 2018 School, family, and community partnerships: Preparing educators and improving schools in School, family, and community partnerships: Preparing educators and improving schools. Routledge.

[25] Lopez ME, Caspe M (2014). Family engagement in anywhere anytime learning. Family InvolvE Net Edu..

[26] Hornby G, Lafaele R (2018). Barriers to parental involvement in education: an update. Educ Rev.

[27] Herodotou C (2018). Young children and tablets: A systematic review of effects on learning and development. J Comput Assist Learn.

[28] Burnett C (2010). Technology and literacy in early childhood educational settings: a review of research. J Early Child Lit.

[29] Laranjeiro D (2021). Development of game-based m-learning apps for preschoolers. Edu Sci..

[30] Papadakis ST, Kalogiannakis M (2017). Mobile educational applications for children. What educators and parents need to know. Inter J Mobile Learn Organ..

[31] Vaiopoulou J, Papadakis S, Sifaki E, Stamovlasis D, Kalogiannakis M (2021). Parents’ perceptions of educational apps use for kindergarten children: Development and validation of a new instrument (PEAU-p) and exploration of parents’ profiles. Behav Sci.

[32] OECD 2020 Strengthening online learning when schools are closed: The role of families and teachers in supporting students during the COVID-19 crisis. Paris Doi: 10.1787/c4ecba6c-en.

[33] Plomp T (2013). Educational Design Research: An Introduction.

[34] J. van den Akker, “Principles and Methods of Development Research,” in *Design Approaches and Tools in Education and Training*, S. Science and B. M. Dorrecht, Eds. 1999, pp. 1–22. doi: 10.1007/978-94-011-4255-7.

[35] Anderson T, Shattuck J (2012). Design-Based Research: A Decade of Progress in Education Research?. Educ Res.

[36] N. Nieveen and E. Folmer, “Formative evaluation in Educational Design Research,” in *Educational Design Research*, Enschede, 2013, pp. 152–169. [Online]. Available: http://international.slo.nl/publications/edr/

[37] Grunau J, Gössling B (2020). Cooperation between research and practice for the development of innovations in an educational design project. Edu Design res.

[38] Ross SM, Din M (2008). Research designs. Handbook of research on educational communications and technology.

[39] Bryman A, Din M (2012). Social research strategies. Social Research Methods.

[40] The Design-Based Research Collective (2003). Design-based research: an emerging paradigm for educational inquiry. Educ Res.

[41] Richey RC, Klein JD, Nelson WA (2004). Developmental Research: Studies of Instructional Design and Development. Development.

[42] Bannan-Ritland B (2003). The Role of Design in Research: The Integrative Learning Design Framework. Educ Res.

[43] Barab S, Squire K (2004). Design-Based Research: Putting a Stake in the Ground. J Learn Sci.

[44] INE, “Anuário Estatístico de Portugal 2016,” Lisboa, 2017.

[45] Grant L (2011). ‘I’m a completely different person at home’: using digital technologies to connect learning between home and school. J Comput Assist Learn.

[46] Knauf H (2016). Interlaced social worlds: exploring the use of social media in the kindergarten the kindergarten. Early Years.

[47] Laranjeiro D, Antunes MJ, Santos P (2017). Development of a multimedia platform for parental involvement in learning of children attending kindergarten - Preliminary Studies”, in INTED2017 - international technology. Edu Develop.

[48] J. Nielsen, “Paper Prototyping: Getting User Data before you code,” *Nielsen Norman Group*, 2003. https://www.nngroup.com/articles/paper-prototyping/

[49] Laranjeiro D, Antunes MJ, Santos P (2018). From Idea to Product – Participation of Users in the Development Process of a Multimedia Platform for Parental Involvement in Kindergarten. Commun Comp inf Sci.

[50] L. Bardin, *Análise de Conteúdo*, Trad. Lisboa, 2004. [Online]. Available: http://books.google.com/books?id=AFpxPgAACAAJ

[51] Wenger E, McDermott R, Snyder WM (2002). Cultivating communities of practice.

[52] Moll LC, Amanti C, Neff D, Gonzalez N (1992). Funds of Knowledge for teaching: using a qualitative approach to connect homes and classrooms. Theory Pract.

[53] Kelly A, Baek J, Lesh R, Bannan-Ritland B, Din M (2008). Enabling Innovations in Education and Systematizing their Impact. Handbook of Design Research Methods in Education - Innovations in Science, Technology.

[54] G. Salmon, *E-moderating*, 2nd ed. Routledge, 2004.

